# Improved GFP Variants to Study Gene Expression in Haloarchaea

**DOI:** 10.3389/fmicb.2019.01200

**Published:** 2019-05-29

**Authors:** Johannes Born, Felicitas Pfeifer

**Affiliations:** Microbiology and Archaea, Department of Biology, Technische Universität Darmstadt, Darmstadt, Germany

**Keywords:** promoter studies, 5′-untranslated region, *Halobacterium*, *Haloferax*, gas vesicle genes

## Abstract

The study of promoter activities in haloarchaea is carried out exclusively using enzymes as reporters. An alternative reporter is the gene encoding the Green Fluorescent Protein (GFP), a simple and fast tool for investigating promoter strengths. However, the GFP variant smRS-GFP, used to analyze protein stabilities in haloarchaea, is not suitable to quantify weak promoter activities, since the fluorescence signal is too low. We enhanced the fluorescence of smRS-GFP 3.3-fold by introducing ten amino acid substitutions, resulting in mGFP6. Using mGFP6 as reporter, we studied six haloarchaeal promoters exhibiting different promoter strengths. The strongest activity was observed with the housekeeping promoters *P_fdx_* of the ferredoxin gene and *P2* of the ribosomal 16S rRNA gene. Much lower activities were determined for the promoters of the p-vac region driving the expression of gas vesicle protein (*gvp*) genes in *Halobacterium salinarum* PHH1. The basal promoter strength dropped in the order *P_pA_*, *P_pO_* > *P_pF_*, *P_pD_*. All promoters showed a growth-dependent activity pattern. The GvpE-induced activities of *P_pA_* and *P_pD_* were high, but lower compared to the *P_fdx_* or *P2* promoter activities. The mGFP6 reporter was also used to investigate the regulatory effects of 5′-untranslated regions (5′-UTRs) of three different *gvp* mRNAs. A deletion of the 5′-UTR always resulted in an increased expression, implying a negative effect of the 5′-UTRs on translation. Our experiments confirmed mGFP6 as simple, fast and sensitive reporter to study gene expression in haloarchaea.

## Introduction

The expression of many haloarchaeal genes depends on the growth phase and environmental conditions such as oxygen availability, salt concentration or temperature (Pfeifer, 2015). Studies by transcriptome or proteome analyses yield global data on gene expression, but the analysis of single genes and promotors is still required to characterize regulatory elements. So far, enzymatic assays were used to study promoter activities in haloarchaea. Commonly applied reporters are the beta-galactosidase BgaH of *Haloferax (Hfx.) alicantei* and the dihydrofolate reductase of *Hfx. volcanii* (Danner and Soppa, 1996; Gregor and Pfeifer, 2001). In *Escherichia coli*, the green fluorescent protein (GFP) is a suitable alternative reporter (Lissemore et al., 2000). Compared to enzymatic assays, GFP-fluorescence based analyses are less time consuming, require no additional substrates and/or cofactors and also no cell lysis. A fusion of the *gfp* reading frame to the promoter under investigation leads to fluorescent *E. coli* cells when the promoter is active. The strength of the GFP signal correlates with the promoter activity and thus with the expression of the gene investigated (Albano et al., 1996, 1998).

Different GFP proteins are used in bacteria and eukaryotes, but in archaea their use is still scarce due to the extreme growth conditions. In the case of haloarchaea the application of GFP is restricted due to the high intracellular salt concentration of 2–5.3 M KCl and a temperature optimum of 42°C (Cormack et al., 1996; Patterson et al., 1997; Nomura and Harada, 1998; Oren, 2002; Enoki et al., 2004; Reuter and Maupin-Furlow, 2004; Robinson et al., 2005; Arpino et al., 2012; Stepanenko et al., 2014). A GFP derivative with an adequate fluorescence in haloarchaea is the short-lived, red-shifted GFP smRS-GFP that has been used to investigate protein degradation in *Hfx. volcanii* (Reuter and Maupin-Furlow, 2004; Schmidt and Pfeifer, 2013). However, the study of weak promoters is not possible since the fluorescence signal is too low.

SmRS-GFP carries the substitutions S65T, Q80R, P99S, M153T, and V163A, improving its solubility and thermostability in the haloarchaeal intracellular milieu (Reuter and Maupin-Furlow, 2004; Baffour-Awuah et al., 2005). The literature on GFP provides additional mutations to improve its properties. The exchange of alanine 206, leucine 221, and phenylalanine 223 by arginine or lysine reduces the dimerization potential of GFP (Zacharias et al., 2002; Enoki et al., 2004; Jackson et al., 2006). Substitutions improving the folding rate and protein stability are S30R, Y39N, S147P, or N149K (Kimata et al., 1997; Pédelacq et al., 2005; Kremers et al., 2011). A substitution with a dramatic effect on GFP fluorescence is F64L, leading to a better maturation at 37°C (Cormack et al., 1996; Scholz et al., 2000). We already introduced the substitution F64L in smRS-GFP, and the resulting mGFP2 shows a 2.5-fold increase in fluorescence (Winter et al., 2018). The mGFP2 variant was used to establish the split-GFP method for haloarchaea and helped to identify several interactions of gas vesicle proteins of *Halobacterium (Hbt.) salinarum* PHH1.

**FIGURE 1:**
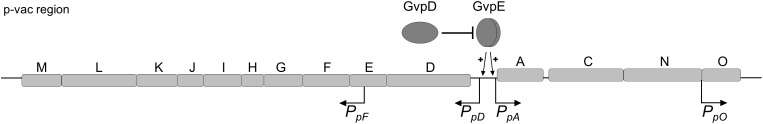
Schematic representation of the p-vac region of *Hbt. salinarum* PHH1. The 14 gas vesicles genes are arranged in two oppositely oriented gene clusters. The expression is driven by the four promoters *P_pA_*, *P_pD_*, *P_pF_*, and *P_pO_*. The activity of *P_pA_* and *P_pD_* is enhanced by the endogenous activator GvpE. In presence of GvpD the amount of GvpE decreases leading to a reduced expression. The p-*gvpACNO*, p-*gvpDE*, and p-*gvpF-M* transcripts contain a 5′-UTR of 20, 72, and 169 nt, respectively.

Gas vesicle formation involves the 14 *gvp* genes arranged as oppositely oriented gene clusters *p-gvpACNO* and *p-gvpDEFGHIJKLM* in the p-vac region (Figure 1; Horne et al., 1991; Englert et al., 1992a). Transcription of the *gvp* genes is controlled by the promoters *P_pA_* and *P_pO_* as well as *P_pD_* and *P_pF_* (Hofacker et al., 2004). *P_p_*_A_ drives the expression of p-*gvpACNO* encoding the two major gas vesicle structural proteins GvpA and GvpC, whereas the *P_pO_* promoter is responsible for the transcription of p-*gvpO* encoding a protein of unknown function (Offner et al., 1996). In the opposite direction, the *P_pD_* promoter drives the expression of *p-gvpDE* encoding the two regulatory proteins GvpE (activator) and GvpD (repressor). GvpE activates the oppositely oriented *P_pA_* and *P_pD_* at high levels (Hofacker et al., 2004). In the presence of GvpD, the GvpE-mediated activation is reduced (Englert et al., 1992b; Zimmermann and Pfeifer, 2003; Schmidt and Pfeifer, 2013). *P_pF_* is responsible for the transcription of p-*gvpFGHIJKLM* encoding the accessory proteins GvpF through GvpM that are required in minor amounts during initial stages of gas vesicle formation (Offner et al., 2000; Winter et al., 2018). The mRNAs of p-*gvpACNO*, p-*gvpDE* and p-*gvpFGHIJKLM* all contain 5′-untranslated regions (5′-UTR), and these 20 nt (p-*gvpA*), 72 nt (p-*gvpD*), and 169 nt (p-*gvpF*) 5′-UTRs might increase the mRNA stability and/or regulate translation initiation.

In the present report we constructed additional variants of smRS-GFP and tested their application to analyze promoter and translational activities in *Hfx. volcanii.* This species is a suitable host since it grows faster than *Hbt. salinarum* and lacks all *gvp* genes. Both haloarchaeal species produce large amounts of carotenoids leading to an autofluorescence; thus, weak promoter activities are difficult to detect by fluorescence. We introduced additional mutations in smRS-GFP and analyzed the fluorescence properties of these variants in *Hfx. volcanii*. The highest fluorescence was observed with mGFP6 harboring ten additional alterations. mGFP6 was used to investigate six haloarchaeal promoters exhibiting weak or strong activities during growth. Due to the fast degradation of mGFP6, we were able to observe growth-dependent expressions. mGFP6 was also used to study the regulatory effects of the three 5′-UTRs on the expression of *gvp* genes.

## Materials and Methods

### Strains and Cultivation Conditions

The *Escherichia coli* strain Top10F’ (Invitrogen, Carlsbad, United States) was grown at 37°C overnight in Luria-Bertani broth. For selection of ampicillin-resistant clones, the medium was supplemented with 100 μg/ml ampicillin. The *Hfx. volcanii* strains used are listed in Supplementary Table S1. *Hfx. volcanii* WFD11 and WR340 were cultivated in medium containing 3 M NaCl, 150 mM MgSO_4_, 50 mM KCl, 10 nM MnCl_2_, 25 mM Tris/HCl pH 7.2, 0.5% (w/v) tryptone, 0.3% (w/v) yeast extract, and 0.02% (w/v) histidine. In case of *Hfx. volcanii* H1424, Hv-Ca medium (Allers et al., 2004) was used, supplemented with thymidine (40 μg/ml) and uracil (50 μg/ml). *Hfx. volcanii* transformants were selected by 6 mg/ml lovastatin (Lam and Doolittle, 1989). Plates with solid media (containing 1.8% [w/v] agar) were incubated in plastic bags at 42°C under humid conditions for 4–5 days.

### Construction of Plasmids for Promoter Studies

For promotor studies, various shuttle plasmids were generated based on pLacZJB18 or pLacZJB18+E (Supplementary Figure S1). Plasmid pLacZJB18 was produced with the NEBuilder^®^ HiFi DNA Assembly Master Mix (New England Biolabs) and carries the *lacZ* gene upstream of the *smRS-gfp* reading frame allowing blue-white selection in *E. coli*. The *lacZ*-*smRS-gfp* fragment is framed by the archaeal L11e rRNA terminator (t.L11e) and a synthetic terminator sequence (Allers et al., 2010) to prevent an unintended read-through of transcripts. This vector enables a replacement of *lacZ* with the promoter sequence of choice *via Nco*I and *Bam*HI restriction sites. Additionally, the *gfp* reporter gene can be exchanged using *Bam*HI and *Kpn*I. For a high copy number in *E. coli*, pLacZJB18 contains the pMB1 origin derivative of pUC19, whereas the pHK2 origin is used for the replication in haloarchaea, resulting in 7–8 copies per genome equivalent (Holmes et al., 1994). As selectable markers served *ampR* (*E. coli*) and the *hmgA* gene (haloarchaea) (Supplementary Figure S1A). To generate pLacZJB18+E including the activator gene c-*gvpE*, the reading frame was amplified by PCR. One of the oligonucleotides used contained the sequence of the *P_fdx_* promoter to drive the expression (Table 1). The *P_fdx_*-*c-gvpE* sequence was inserted behind t.L11e in pLacZJB18 using *Nhe*I and *Xba*I. In order to exclude an inhibition of *c-gvpE* expression by t.L11e, a 587 nt non-coding sequence was inserted between the terminator and *P_fdx_* (Supplementary Figure S1B). The *c-gvpE* gene can be replaced using *Spe*I and *Xba*I, while substitution of *P_fdx_* by another promoter sequence could be performed *via Nhe*I and *Spe*I.

**Table 1:** Oligonucleotides used in this study.

Name	Sequence (5′–3′)
**Oligonucleotides for construction of pLacZJB18**	
pLacJB18_1	CGGTCATCGGAACCCCTATTTGTTTATTTTTCTAAATACATTCAAATATGTATCCGCTC
pLacJB18_2	CCATGGTTTATCTTCCGCTTCCTCGCTCACTGACT
pLacJB18_3	AAGCGGAAGATAAACCATGGATTAAGCTTCCCGGG
pLacJB18_4	GGCGCGTCTCTCCAGGTAGCGAAAGCCATTTTTTG
pLacJB18_5	GCTACCTGGAGAGACGCGCCCGCTGATCCTTTGCG
pLacJB18_6	CGGTCGGTAACGCGCCGAAAAATGCGATGGTCCAG
pLacJB18_7	TTTCGGCGCGTTACCGACCGAGTTCGGCGTGGGCG
pLacJB18_8	CGAGTCGCCGACGTTCGACCCCGACGCGGGAGGGC
pLacJB18_9	GGTCGAACGTCGGCGACTCGACCTCGAAGTGGTCG
pLacJB18_10	AATAGGGGTTCCGATGACCGGCTCGTCCACGTCGA
**Oligonucleotides for construction of pLacZJB18+E. NheI and XbaI recognition site are highlighted in bold.**	
FDX-gvpE_fwd	ATAGA**GCTAGC**CGGGCTTTCGTGGCAGTACGCTGGCCCGAACAGCAACTACTATGCGTTCGGAAGCCGAACTCTGCAGACTAGTATGGACGACCTCTTAGCGGAGC
FDX-gvpE_rev	GTGTATCTAGAATCACTCATCCTGGGGGCTGTG
**Oligonucleotides for insertion of promoter sequence in pLacZJB18. NcoI and BamHI overhangs are highlighted in bold. AscI recognitionsite is indicated by small letters.**	
FDX_fwd	**CATGG**ggcgcgccCGGGCTTTCGTGGCAGTACGCTGGCCCGAACAGCAACTACTATGCGTTCGGAAGCCGAACTCTGCAGTG**G**
FDX_rev	**GATCC**CTGCAGAGTTCGGCTTCCGAACGCATAGTAGTTGCTGTTCGGGCCAGCGTACTGCCACGAAAGCCCGggcgcgcc**C**
P2_fwd	**CATGG**ggcgcgccCGATGCCCTTAAGTACAACAGGGTACTTCGGTGGAATGCGAACGACA**G**
P2_rev	**GATCC**TGTCGTTCGCATTCCACCGAAGTACCCTGTTGTACTTAAGGGCATCGggcgcgcc**C**
PpA_fwd	**CATGG**ggcgcgccTCATTACAGGAGACATAACGACTGGTGAAACCATACACATCCTTATGTGATGCCCGAGTATAGTTAGAG##TGGGTTAATCCCAGATCACCAATGGCGCAACCAGAT**G**
PpA_rev	**GATCC**ATCTGGTTGCGCCATTGGTGATCTGGGATTAACCCATCTCTAACTATACTCGGGCATCACATAAGGATGTGTATGGTTTCACCAGTCGTTATGTCTCCTGTAATGAggcgcgcc**C**
PpO_fwd	**CATGG**ggcgcgccAAATAGAATCCGCGATCGACGACATGGAAGTCGCCCTTTCTTAAGATCCGGGGTCTCTACATAGAAGCATGGCAGATCCAGCA**G**
PpO_rev	**GATCC**TGCTGGATCTGCCATGCTTCTATGTAGAGACCCCGGATCTTAAGAAAGGGCGACTTCCATGTCGTCGATCGCGGATTCTATTTggcgcgcc**C**
PpA_Δ5_fwd	**CATGG**ggcgcgccTCATTACAGGAGACATAACGACTGGTGAAACCATACACATCCTTATGTGATGCCCGAGTATAGTTAGAGATGGATGGCGCAACCAGAT**G**
PpA_Δ5_rev	**GATCC**ATCTGGTTGCGCCATCCATCTCTAACTATACTCGGGCATCACATAAGGATGTGTATGGTTTCACCAGTCGTTATGTCTCCTGTAATGAggcgcgcc**C**
PpD_Δ5_fwd	**CATGG**ggcgcgccATGGTTTCACCAGTCGTTATGTCTCCTGTAATGAGTCGTACTTCTAAGTACGGAGAGTGTAAAGCTTCTTAGACatgagttcacccaat**G**
PpD_Δ5_rev	**GATCC**ATTGGGTGAACTCATGTCTAAGAAGCTTTACACTCTCCGTACTTAGAAGTACGACTCATTACAGGAGACATAACGACTGGTGAAACCATggcgcgcc**C**
PpF_Δ5_fwd	**CATGG**ggcgcgccTCTCCGGCGGCTGTTTGGGGCAGACCTGAGTCCGGGTACAGTATACCCGCATTTAAATGACCTTGCAGTCGAAGGTGTACTTGAATGACTGAGAACCTA**G**
PpF_Δ5_rev	**GATCC**TAGGTTCTCAGTCATTCAAGTACACCTTCGACTGCAAGGTCATTTAAATGCGGGTATACTGTACCCGGACTCAGGTCTGCCCCAAACAGCCGCCGGAGAggcgcgcc**C**
**Oligonucleotides for amplification of promoter sequence. NcoI and BamHI recognition site are highlighted in bold. AscI recognition site isindicated by small letters.**	
PpD_fwd	TATAT**CCATGG**ggcgcgcctATGGTTTCACCAGTCGTTATGTC
PpD_rev	TGATT**GGATCC**ATTGGGTGAACTCATTACTTCTCTC
PpF_fwd	TATAT**CCATGG**ggcgcgccTCTCCGGCGGCTGTTTG
PpF_rev	TGATT**GGATCC**TAGGTTCTCAGTCATTGGTCTCTCTTCC
**Oligonucleotides for amplification of reporter genes. BamHI and KpnI recognition site are highlighted in bold.**	
eyfp_fwd	TACTA**GGATCC**ATGGTGAGCAAGGGCGAGGAGC
eyfp_rev	ATCTA**GGTACC**GCGGCCGCTTATTACTTGTACAGCTCGTCCATGCCGAGAGTGATCC
ecfp_fwd	TACTA**GGATCC**ATGGTGAGCAAGGGCGAGGAGCT
ecfp_rev	AGCTA**GGTACC**GCGGCCGCTTATTACTTGTACAGCTCGTCCATGCCGAGAGTGA
mTagBFP_fwd	TACTA**GGATCC**ATGAGCGAACTGATCAAAGAGAACAT
mTagBFP_rev	ATCTA**GGTACC**GCGGCCGCTTATTAATTCAGTTTATGACCCAGCTTGCTAG
SYFP2_fwd	TACTA**GGATCC**ATGGTTAGCAAGGGCGAAGAACTTTTT
SYFP2_rev	ATCTA**GGTACC**GCGGCCGCTTATTATTTATACAGCTCATCCATACCCAGGGTAATAC
sfGFP_fwd	TACTA**GGATCC**ATGCGTAAAGGCGAAGAGCTGTT
sfGFP_rev	ATCTA**GGTACC**GCGGCCGCTTTGTACAGTTCATCCATACCATGCGTG
**Oligonucleotides for mutagenesis of smRS-GFP. Nucleotide substitutions are indicated in small letters.**	
mGFP_1_fwd	GATGCAACAaACGGAAAACTTACCCTT
mGFP_1_rev	ACCTTCACCCTCTCCcCTGACAGA
mGFP_2_fwd	GTCACTACTcTCACTTATGGTGTTCGT
mGFP_2_rev	AGTGTTGGCCATGGAACAGGTA
mGFP_3_fwd	GTGTTCAATGCTTTgCAAGATACCCA
mGFP_3_rev	CATAAGTGAgAGTAGTGACAAGTGTTGGC
mGFP_4_fwd	CAACcCCCACAAaGTATACATCACG
mGFP_4_rev	TAGTTGTATTCCAACTTGTGGCCGA
mGFP_5_fwd	GAAAGATCCCAACGAAAAGAGAGA
mGFP_5_rev	GAAAGcttAGATTGTGTGGACAGGTA
mGFP_6_fwd	GAGcgTGTAACtGCTGCTGGGATTA
mGFP_6_rev	tttAAGgACCATGTGGTCTCTCTTTTCG

To fuse promoter sequences up to 120 bp with the *gfp* reporter gene, two complementary oligonucleotides covering the entire promoter sequence were synthesized and hybridized to yield a double-stranded fragment with nucleotide overhangs of *Nco*I and *Bam*HI. After 5′-phosphorylation, the promoter fragment was inserted in pLacZJB18 or one of its derivatives, *via Bam*HI and *Nco*I. Promoter sequences exceeding 120 bp were amplified by PCR, and the resulting fragments were inserted in pLacZJB18. For the integration of different *gfp* reporter genes served *Bam*HI and *Kpn*I. In case of plasmids used to quantify promoter activities, an *Asc*I recognition site was present in the oligonucleotide upstream of the *Nco*I-site and served for analytical digests. All constructs were verified by DNA sequencing (Eurofins genomics). *Hfx. volcanii* was transformed as described by Pfeifer and Ghahraman (1993) and the transformants were controlled by PCR and DNA sequencing (Eurofins genomics) for uptake of the desired plasmid. All oligonucleotides used for these constructions are listed in Table 1.

### Detection and Quantification of Fluorescence *in vivo*

For the detection of the smRS-GFP fluorescence in colonies of *Hfx. volcanii*, the cells were grown for 6 days on agar plates and then inspected using a fluorescence binocular. Colonies of the wild type served as negative control. To quantify promoter activities *in vivo*, the fluorescence was measured in *Hfx. volcanii* liquid cultures. For that, 20 ml cultures were inoculated to OD_600_ 0.02 and cultivated at 42°C and 180 rpm to an optical density of OD_600_ 0.3, 0.6, 1.2, and 3.0. This corresponded to an incubation time of 16, 24, 36, and 48 h. Cells of a 1 ml sample of each culture were harvested by centrifugation at 5,000 ×*g* for 5 min at 20°C. The sediment was washed twice with salt water (3 M NaCl, 150 mM MgSO_4_, 50 mM KCl, 0.02% [w/v] histidine) and then resuspended in 2 ml salt water. Fluorescence measurements were conducted with a Fluorolog FL3-22 (Horiba Jobin Yvon) at 25°C with an excitation wavelength of 488 nm (slit 5 nm) and an emission wavelength of 509 nm (slit 5 nm). As integration time 0.5 s was selected. In case of different fluorescent proteins, the fluorescence was determined at the respective optimal excitation and emission wavelength. The optical density of each culture was determined to normalize the fluorescence intensity. The autofluorescence of the wild type was subtracted from the data. Each experiment was carried out in triplicates with three biological replicates.

### Fluorescence Microscopy

*Hfx. volcanii* cells were visualized by brightfield and fluorescence microscopy using a Leica TCS SP5 II confocal microscope in combination with Leica application suite software. For this, cells of a 2 ml sample of culture were harvested by centrifugation at room temperature at 8,000 rpm for 2 min, the supernatant was removed and the cell sediment resuspended in salt water. The detection of the fluorescence of 20 μl of the suspension was done by an excitation of 488 nm and an emission of 510–580 nm. For image processing the software Fiji was used.

### Mutation of smRS-GFP

To improve the fluorescence of smRS-GFP, different substitutions were introduced in the gene by site-directed mutagenesis. The mutagenesis was performed by amplification of the complete vector using two oligonucleotides flanking each other at the 5′ ends. One of the oligonucleotides contained one to three nucleotide substitutions leading to the desired mutations in the *smRS-gfp* gene. The oligonucleotides used are listed in Table 1. After PCR the template vector was degraded with *Dpn*I at 37°C for 1 h, and the amplified PCR-fragments were purified. The 5′ ends of the linear PCR products were phosphorylated by T4-polynucleotide kinase and ligated by T4-Ligase in one step (1 h, 37°C) to generate the circular plasmid. *E. coli* Top10 was transformed and the plasmids obtained from the transformants were controlled by DNA sequencing (Eurofins).

### Western Analysis

The presence of GvpE in transformants harboring derivatives of pLacZJB18+E was determined by Western analysis. Transformants were grown in 50 ml cultures to OD_600_ 1.2, harvested by centrifugation (2,000 ×*g*, 45 min, 4°C) and the sediment was resuspended in 2–3 ml lysis buffer (2.5 M KCl, 50 mM MgCl_2_, 1 mM EDTA, 5% [v/v] glycerol, 50 mM Tris-HCl pH 8.0). The cells were disrupted by sonication on ice (2 × 5 min, Branson sonifier 250, 3 mm disruptor horn) and the suspension centrifuged at 2,000 ×*g* for 45 min at 4°C. To remove the high amount of salt the suspension was dialyzed against 10 mM Tris-HCl pH 7.2 for 12 h. After dialysis, 20 μg of the total protein were separated by SDS-PAGE (Schägger and von Jagow, 1987) and transferred to a PVDF membrane (Roti^®^-Fluoro PVDF, Carl Roth) using the PerfectBlue^TM^ “Semi-Dry”-Blotter. The membrane was subsequently incubated for 1 h at 37°C, reactivated in 100% methanol, washed for 4 min with PBS (1.37 M NaCl, 27 mM KCl, 100 mM Na_2_HPO_4_, 20 mM KH_2_PO_4_, pH 7.4) and blocked for 1 h with Odyssey Blocking Buffer (LI-COR). After incubation with the GvpE antiserum in Odyssey Blocking Buffer overnight, the membrane was washed four times for 5 min with PBS + 0.1% (v/v) Tween^®^ 20 and incubated with the secondary antibody IRDye 800CW (LI-COR) coupled with a fluorophore detectable at 800 nm. The detection was done with the Odyssey Fc Imager (LI-COR).

## Results

### Fluorescence of smRS-GFP in Three *Hfx. volcanii* Strains at Various Temperatures

**FIGURE 2:**
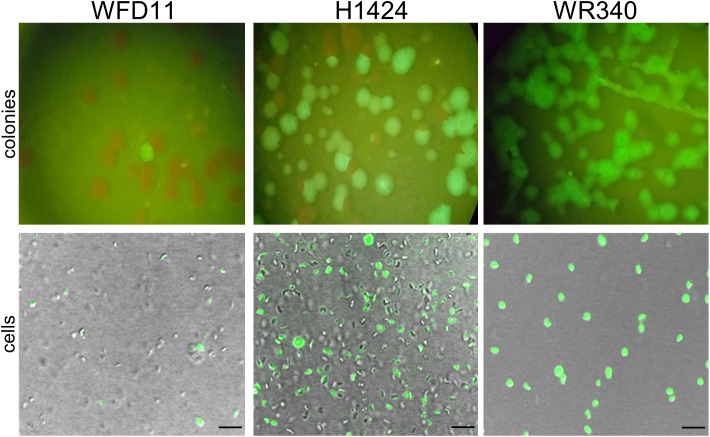
Expression of *smRS-gfp* in three different *Hfx. volcanii* strains. The transformants of *Hfx. volcanii* WFD11, H1224, and WR340 carry pP_fdx_JB18 harboring the *smgRS-gfp* reporter under control of *P_fdx_*. The fluorescence of the colonies was visualized under a fluorescence binocular (with blue light) and the fluorescence of cells by confocal laser scanning microscopy. Scale bars equal 10 μm in each case.

The expression of *smRS-gfp* was analyzed in the three *Hfx. volcanii* strains WR340, WFD11, and H1424 available in our laboratory (Cline et al., 1989; Bitan-Banin et al., 2003; Stroud et al., 2012). The *lacZ* gene of plasmid pLacZJB18 was exchanged for the ferredoxin promoter *P_fdx_* of *Hbt. salinarum* (Pfeifer et al., 1993). The three strains were transformed with the resulting plasmid pP_fdx_JB18 and the fluorescence of the colonies was inspected on agar plates illuminated with blue light (Figure 2). The cells were also analyzed by fluorescence microscopy. Only a small fraction of colonies (WFD11) or about half of the colonies (H1424) exhibited a fluorescence, but every colony of WR340 was fluorescent. *Hfx. volcanii* WR340 appeared to be more stable in the expression of *smRS-gfp* and was used for all further experiments. To investigate the influence of the temperature on the *smRS-gfp* expression, pP_fdx_JB18 WR340 transformants were grown at 37, 42°C, or 45°C to OD_600_ 0.6. The cells grown at 42°C reached a 1.5- to 1.9-fold higher fluorescence (367 ± 34 ×10^3^ AU) than those cultured at 37°C (188 ± 17 ×10^3^AU) or 45°C (242 ± 39 ×10^3^ AU). Thus, all further growth experiments were performed at the optimal growth and folding temperature of 42°C.

### Comparison of smRS-GFP With Other Fluorescent Proteins in *Hfx. volcanii*

**FIGURE 3:**
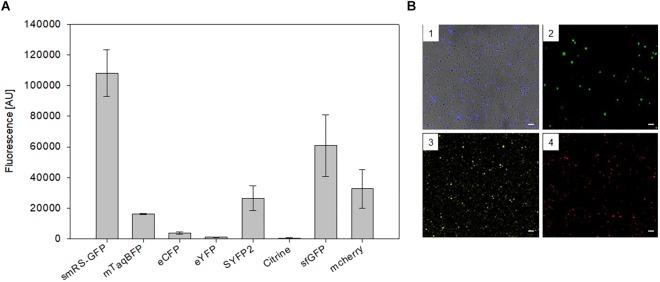
Expression of different fluorescent proteins in *Hfx. volcanii* WR340. **(A)** The expression of the fluorescent protein genes was driven by the strong *P_fdx_* promoter. The transformants were grown to OD_600_ 0.6 at 42°C. The fluorescence is given in arbitrary units (AU). The autofluorescence of *Hfx. volcanii* was subtracted from each value. All experiments were performed in triplicates with three different biological samples. **(B)** Fluorescence microscopy of *Hfx. volcanii* WR430 expressing the reading frames encoding mTagBFP (1), mGFP2 (2), SYFP2 (3), and mCherry (4). Scar bars equal 10 μm in each case.

To test other available fluorescent proteins, the *smRS-gfp* reading frame of vector pP_fdx_JB18 was exchanged with the reading frames encoding mTagBFP (blue), eCFP (cyan), sfGFP (superfolder GFP, green), eYFP, Citrine as well as SYFP2 (yellow), or mCherry (red). The respective *Hfx. volcanii* WR340 transformants were grown to OD_600_ 0.6 at 42°C and their fluorescence was determined. The highest fluorescence (108,000 AU) was observed with the original smRS-GFP, but the fluorescence of sfGFP, mCherry, SYFP2, and mTagBFP were well detectable (between 16,000 and 60,000 AU) (Figure 3A). Cells were also detectable by fluorescence microscopy (Figure 3B). However, the fluorescence of eCFP, eYFP or Citrine in the transformants was very low and difficult to detect.

### Mutation of smRS-GFP to Enhance the Fluorescence

**FIGURE 4:**
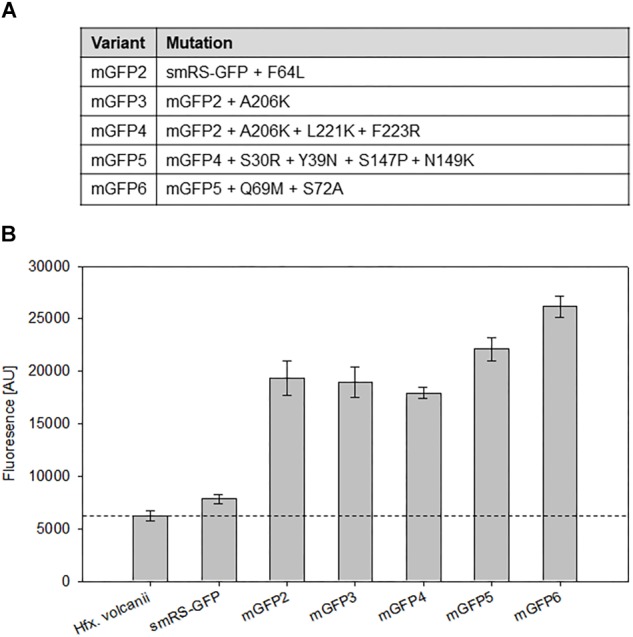
Point mutations in smRS-GFP. **(A)** Mutations in smRS-GFP to yield the variants mGFP2 through mGFP6. The selected substitutions influence maturation at 37°C, the tendency of self-aggregation, the folding rate, or protein stability. **(B)** Fluorescence of smRS-GFP variants *in vivo*. Fluorescence of wild type cells and transformants carrying the reading frame encoding one of the variants under control of the weak *P_pD_* promoter. The cells were grown for 24 h to OD_600_ 0.6 at 42°C. The fluorescence is given in arbitrary units (AU). Each experiment was performed in triplicates of three different biological samples.

To investigate whether smRS-GFP allows the analysis of weak promoter activities, the promoter *P_pD_* (plus 5′-UTR) of the p-vac region was chosen. The respective plasmid pP_D_JB18 (*smRS-gfp* under control of *P_pD_*) was constructed and investigated in *Hfx. volcanii* WR340 transformants. The fluorescence of cells in the early exponential growth phase (OD_600_ 0.3) was hardly above the autofluorescence of *Hfx. volcanii* (Figure 4B). In order to generate smRS-GFP variants exhibiting a stronger fluorescence, additional mutations (F64L, A206K, L221K, F223R, S30R, Y39N, S147P, N149K, and S72A) known to improve the GFP properties in *E. coli* (Kimata et al., 1997; Pédelacq et al., 2005; Kremers et al., 2011) were introduced individually or in combination to increase its brightness in haloarchaea (Figure 4A). The fluorescence of the respective *Hfx. volcanii* transformants harboring these variants was measured in the early exponential growth phase at OD_600_ 0.3 (Figure 4B). The mGFP-F64L mutant (mGFP2) yielded a 2.5-fold higher fluorescence, similar to mGFP3 and mGFP4, whereas variant mGFP5 showed a 2.9-fold higher fluorescence compared to smRS-GFP. The strongest fluorescence (3.3-fold enhanced) was observed with mGFP6 carrying all ten substitutions (Figure 4B).

### Quantification of Promoter Activities Using mGFP6

**FIGURE 5:**
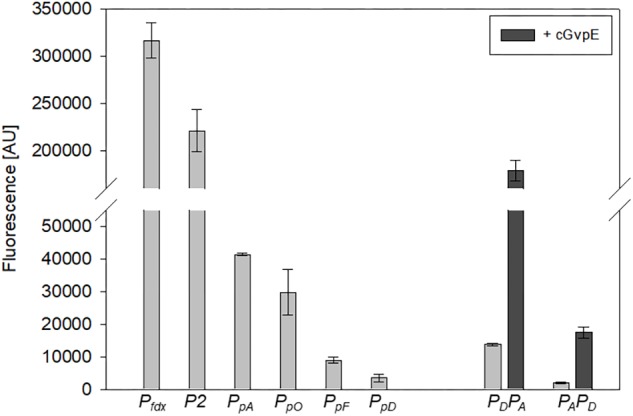
Activities of the six different haloarchaeal promoters. The activity was quantified at OD_600_ 0.6 (42°C). The fluorescence is given in arbitrary units (AU) (see Table 2). The autofluorescence of *Hfx. volcanii* was subtracted from the values. Each experiment was performed in triplicates of three different biological samples. The *P_pA_* and *P_pD_* activities induced by cGvpE are shown on the right (OD_600_ 0.6). The *P_D_P_A_* and *P_A_P_D_* constructs used for these experiments include the entire *P_pA_-P_pD_* promoter region. For more information, see text.

The strength of six different haloarchaeal promoters was determined using mGFP6 as reporter. The two house-keeping gene promoters *P_fdx_* (ferredoxin gene), *P2* (ribosomal 16S rRNA gene) and the four promoters of the gas-vesicle encoding p-vac region of *Hbt. salinarum* PHH1 were investigated. In case of the gas vesicle promoters, the complete promoter sequence up to the first 15 nt (including the ATG start) of the corresponding *gvp* gene were fused to *mgfp6* lacking its AUG start codon. Samples of the respective *Hfx. volcanii* transformants were taken at OD_600_ 0.3, 0.6, 1.2, and 3.0, i.e., after 16, 24, 36, or 48 h of growth, and analyzed for fluorescence (Table 2; see Figure 5). The *P_fdx_*-*mgfp6* transformants yielded the highest fluorescence in the exponential growth phase, and the activity decreased approximately twofold during stationary growth (Table 2). These results implied that the product ferredoxin is mainly required during exponential growth. *P2* was constitutively active in the exponential growth phase, but at a slightly lower level compared to *P_fdx_*. In the stationary growth phase, the *P2*-driven expression was comparable to that of *P_fdx_* (Table 2). The much lower basal activity of *P_pA_* was highest in the early exponential growth phase, a reduced fluorescence was observed in late exponential and early stationary growth, and the fluorescence was again higher in the late stationary growth phase (Table 2). *P_pO_* yielded a high fluorescence in early exponential growth, which was reduced during exponential growth, increased and dropped down again in the late stationary phase (Table 2). In contrast, *P_pD_* and *P_pF_* yielded a 3- to 5-fold lower fluorescence compared to *P_pA_* and *P_pO_*. Both showed their highest value in the early exponential growth phase, followed by a 3- to 4.5-fold reduction up to the stationary growth phase (Table 2). Overall, mGFP6 allowed the detection of all these promoter activities and uncovered different patterns of promoter activity during growth.

**Table 2:** Promoter activities throughout the growth.

Promoter		Basal activity/Fluorescence [AU] ×10^3∗^			
	OD_600_	0.3	0.6	1.2	3.0
*P_fdx_*		318 ± 9	316 ± 18	195 ± 16	159 ± 3
*P2*		212 ± 11	221 ± 22	216 ± 11	162 ± 12
*P_pA_*		69 ± 4	41 ± 0.4	36 ± 4	61 ± 4
*P_pO_*		99 ± 7	30 ± 7	79 ± 1	30 ± 6
*P_pF_*		30 ± 5	9 ± 1	8 ± 0.4	6 ± 0.03
*P_pD_*		18 ± 3	4 ± 1	3 ± 0.1	1 ± 0.01
*P_pA_* Δ5′UTR_A_		101 ± 11	101 ± 8	36 ± 4	67 ± 2
*P_pD_* Δ5′UTR_D_		69 ± 6	25 ± 6	30 ± 3	15 ± 3
*P_pF_* Δ5′UTR_F_		53 ± 8	24 ± 1	23 ± 2	6 ± 0.2

*^∗^Three biological and three technical replicates.*

### Activation of *P_pA_* and *P_pD_* by GvpE

The GvpE-mediated activation of the two promoters *P_pA_* and *P_pD_* was investigated using the vector construct pLacZJB18+E. The plasmid harbors the c-*gvpE* reading frame encoding the activator of the c-vac region of *Hbt. salinarum* PHH1 expressed under the control of the strong *P_fdx_* promoter (Supplementary Figure S1B). The cGvpE protein was used for these studies because of its higher activation capability compared to pGvpE of the p-vac region (Gregor and Pfeifer, 2001). The *lacZ* reading frame was substituted by the entire *P_pD_-P_pA_* promoter region. The resulting plasmid pP_D_P_A_JB18+E (designated P_D_P_A_+E) served to investigate the activation of *P_pA_*, whereas pP_A_P_D_JB18+E (P_A_P_D_+E) was used to investigate the GvpE-activation of *P_pD_*. Construct P_D_P_A_+E contained the first 15 nt of *gvpA* fused to *mgfp6* (i.e., *gfp* under *P_A_* promoter control) and in the opposite direction the entire DNA sequence up to the first 15 nt of the *gvpD* reading frame. Construct P_A_P_D_+E contained the *mgfp6* reading frame fused to the first 15 nt of *gvpD* (*gfp* under *P_D_* promoter control), and in addition all sequences up to the first 15 nt of *gvpA*. Western analysis performed with total proteins of the *P_D_P_A_*+E or *P_A_P_D_*+E transformants using an antiserum raised against cGvpE yielded the presence of GvpE in both cases (Supplementary Figure S2). To determine the basal activities of *P_pA_* or *P_pD_*, similar vectors but lacking c*-gvpE* were used (designated P_D_P_A_ and P_A_P_D_). Based on the high activity of *P_pA_* and *P_pD_* in the exponential growth phase, the fluorescence of the respective *Hfx. volcanii* transformants was analyzed in cells grown to OD_600_ 0.6 at 42°C. The fluorescence of the *P_D_P_A_* transformants (basal *P_pA_* activity) was almost sevenfold higher than the signal of the *P_A_P_D_* transformants (basal *P_pD_* activity), underlining that the *P_pA_* promoter is stronger than *P_pD_* (Figure 5, right side; Table 3). The GvpE-mediated activation resulted in a much higher fluorescence: *P_D_P_A_*+E transformants were 13-fold enhanced in fluorescence compared to *P_D_P_A_* transformants, and the fluorescence of *P_A_P_D_*+E transformants was 8-fold enhanced compared to *P_A_P_D_* (Figure 5 and Table 3). In summary, the expression derived from the cGvpE-induced *P_pA_* promoter was much higher compared to the induced expression derived from *P_pD_*.

**Table 3:** GvpE-induced promoter activities^∗^.

Promoter	Basal activity fluorescence [AU] × 10^3^	cGvpE induced activity fluorescence [AU] × 10^3^	Induction
*P_pD_P_pA_*	14 ± 0.5	179 ± 11	13-fold
*P_pA_P_pD_*	2 ± 0.1	15 ± 1.7	8-fold

*^∗^Three biological and three technical replicates.*

### Effect of 5′-UTRs on *gvp* Translation

**FIGURE 6:**
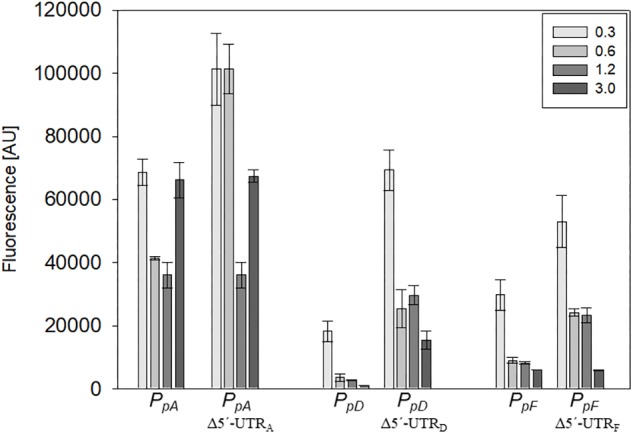
Effect of the 5′-UTR on the expression during growth. The effect of the 5′-UTR_A_, 5′-UTR_D_, and 5′-UTR_F_ on the expression of the corresponding mRNA was quantified throughout growth. The fluorescence of the transformants measured at OD_600_ 0.3, 0.6, 1.2, and 3.0 (42°C) is given in arbitrary units (AU). The autofluorescence of *Hfx. volcanii* was subtracted from each value. Each experiment was performed in triplicates of three different biological samples.

The influence of the 5′-untranslated regions of the p-vac mRNAs p-*gvpACNO* (5′-UTR_A_), p-*gvpDE* (5′-UTR_D_) and p-*gvpFGHIJKLM* (5′-UTR_F_) on translation was investigated using mGFP6 as reporter. The corresponding promoter sequences (including the first 15 nt of the respective reading frame, but lacking the 5′-UTR) were fused to *mgfp6* and the fluorescence was determined in *Hfx. volcanii* transformants at OD_600_ 0.3, 0.6, 1.2, and 3.0 (Figure 6). In each case, lack of the 5′-UTR resulted in a higher fluorescence, especially during the exponential growth phase, suggesting a negative influence of the 5′-untranslated leader regions on translation. The highest fluorescence occurred with Δ5′-UTR_A_ transformants, followed by Δ5′-UTR_D_ and Δ5′-UTR_F_ transformants (Table 2). The strongest effect was observed with Δ5′-UTR_D_ leading up to a sixfold increase in fluorescence in exponential growth. During late exponential growth phase, the fluorescence of the Δ5′-UTR_D_ transformants dropped down, but was still higher than in the presence of the 5′-UTR_D_ (Figure 6 and Table 2). Thus, all three 5′-UTRs significantly reduce the translation of the respective *gvp* mRNA.

## Discussion

To study the activity of weak haloarchaeal promoters, smRS-GFP was altered by additional mutations to improve its brightness. The best variant obtained was mGFP6 containing ten additional mutations and offering a 3.3-fold higher fluorescence signal in *Hfx. volcanii* compared to smRS-GFP. Using mGFP6 as reporter, six haloarchaeal promoters ranging from weak to high activities were investigated, and the activities were also quantified throughout growth. In addition, mGFP6 was used to study the effect of 5′-untranslated regions (5′-UTRs) on the translation of transcripts derived from the p-vac region.

### mGFP6 Is Suitable to Study Weak Promotor Activities

The high salt concentration in the haloarchaeal cytoplasm and growth temperatures above 45°C restrict the GFP fluorescence (Cormack et al., 1996; Nomura and Harada, 1998; Enoki et al., 2004; Stepanenko et al., 2014). The soluble smRS-GFP shows a sufficient signal to investigate protein degradation in haloarchaea, but the signal is too low to quantify weak promoter activities. Several substitutions were introduced in smRS-GFP that are known to improve its maturation at higher temperature, reduce protein self-aggregation and increase folding as well as protein stability (Cormack et al., 1996; Zacharias et al., 2002; Enoki et al., 2004; Jackson et al., 2006). The F64L substitution strongly increases the maturation rate in *E. coli* (Cormack et al., 1996; Scholz et al., 2000). Indeed, the resulting mGFP2 showed a 2.5-fold enhanced fluorescence compared to the initial signal in *Hfx. volcanii* (Figure 4B). The high salt concentrations in the haloarchaeal cytoplasm of 2–5 M KCl may limit the fluorescence of GFP by an increased tendency to self-aggregate or alterations of the protein structure (Deschamps et al., 1995; Ishii et al., 2007). The substitutions A206K, F221K, and F223R were inserted individually or in combination in mGFP2 to reduce its dimerization potential (Zacharias et al., 2002; Enoki et al., 2004; Jackson et al., 2006). However, the mutation A206K (mGFP3) as well as A206K, F221K, and F223R (mGFP4) did not result in an increased fluorescence compared to mGFP2 (Figure 4B).

A most widely used GFP variant in *E. coli* and mammalian cells is sfGFP, whose good folding and stability properties are mainly based on the two substitutions S30R and Y39N (Pédelacq et al., 2005). Other mutations known to improve folding, maturation and protein stability at higher temperatures are the substitutions N149K and S147P (Kimata et al., 1997; Tsien, 1998; Cubitt et al., 1999; Baffour-Awuah et al., 2005; Pédelacq et al., 2005; Choi et al., 2017). The implementation of these four mutations into mGFP4 (to yield mGFP5) led to a 1.2-fold increase in fluorescence. The S72A mutation improves GFP stabilization and folding in *E. coli* (Cubitt et al., 1999), particularly when combined with F64L, S65T and N149K (Teerawanichpan et al., 2007). The resulting mGFP6 showed the strongest fluorescence (3.3-fold increase) compared to smRS-GFP and a 1.2-fold higher signal than mGFP5 in haloarchaea (Figure 4B). It should be noted that mGFP6 carries at position 69 a methionine instead of a glutamine due to a PCR error. The Q69M mutation is known to increase photostability and reduces the sensitivity to chloride ions and low pH values in yellow fluorescent proteins, YFP (Griesbeck et al., 2001). However, the chromophore structure of YFP is not comparable to GFP and the substitution Q69M might not have any effect (Tsien, 1998). Taken together our results suggest that the stabilizing substitutions play a much larger role in the improvement of smRS-GFP brightness in haloarchaea than mutations that might affect its solubility and self-aggregation. The mGFP6 signal finally obtained was high enough to quantify weak promoter activities in haloarchaea.

### Promoter Activities Are Growth-Phase Dependent

To evaluate mGFP6 as a fast and easy alternative to an enzymatic assay we expressed the *mgfp6* reading frame under the control of different promoters and quantified the signal in *Hfx. volcanii* transformants. We selected the house-keeping promoter *P_fdx_* of the ferredoxin gene, the *P2* promoter of ribosomal 16S rRNA gene, and for a moderate to very weak expression *P_pA_*, *P_pD_*, *P_pF_*, and *P_pO_* of the p-vac region of *Hbt. salinarum*. The highest fluorescence was obtained with *P_fdx_*_,_ followed by *P2* (Figure 5). In the case of *P_fdx_*, a constantly high fluorescence signal was observed throughout growth, and a decrease occurred at the beginning of the stationary growth phase. Ferredoxin serves as major electron carrier in haloarchaea and especially in the decarboxylation of α-keto acids (Kerscher and Oesterhelt, 1977; Pfeifer et al., 1993; Falb et al., 2008). The dominant production of *fdx* mRNA during the exponential growth phase is due to the use of ferredoxin as electron transport system in the metabolism of haloarchaea. In contrast, the *P2* promoter showed a constantly high activity throughout growth (Table 2), suggesting that the 16S rRNA is continuously produced (Reuter and Maupin-Furlow, 2004).

In contrast, the promoters *P_pA_*, *P_p_*_O_, *P_pD_*, and *P_pF_* of the p-vac region exhibited much lower activities and also varied in activity throughout growth (Figure 5). Two of these, *P_pA_* and *P_pD_*, are induced by the endogenous activator GvpE (Hofacker et al., 2004). The basal activity of *P_pA_* yielded the highest fluorescence in the early exponential growth phase, and the fluorescence was reduced up to the early stationary growth phase, but increased again in the late stationary growth phase (Table 2). These results were consistent with previous observations using the haloarchaeal beta-galactosidase BgaH as reporter (Hofacker et al., 2004). *P_pA_* drives the transcription of *p-gvpACNO*, leading to large amounts of *p-gvpA* mRNA and minor amounts of *p-gvpAC*, *p-gvpACN*, and *p-gvpACNO* transcripts (Offner et al., 1996). The promoter is also induced by the endogenous activator protein GvpE and leads to the formation of the major gas vesicle structural proteins GvpA and GvpC. The activity of *P_pO_* resulted in a leaderless p-*gvpO* transcript. The promoter *P_pO_* was characterized for the first time, since an activity was not detectable when measured with BgaH as reporter (Hofacker et al., 2004). The reason could be a loss of function of BgaH due to the N-terminal fusion of the first five amino acids of pGvpO (MADPA). The highest activity of *P_pO_* was observed in the early growth phase, the activity decreased during exponential growth, increased at the end of this growth phase and was reduced again in the stationary growth phase (Table 2). The reason for this behavior is not known.

The *P_pD_* promoter exhibited the lowest basal activity determined here. Its highest basal activity was observed in the early exponential growth phase, but then the activity dropped continuously down (Table 2). In *Hbt. salinarum*, the *P_pD_* promoter is activated by GvpE, leading to a much higher expression of the two regulatory proteins GvpD and GvpE. In previous Northern analyses performed with total RNA of *Hbt. salinarum* PHH1, the p-*gvpDE* mRNA was observed in the stationary growth phase only, suggesting that *P_pD_* is active in the late growth phase only. A reason for this difference could be undetectable amounts of transcripts in the early growth phase due to the weak activity of *P_pD_*, whereas GvpE-activation of *P_pD_* leads to a detectable amount of p-*gvpDE* mRNA in the stationary phase. Our analysis on the activity of *P_pA_* and *P_pD_* in the presence of cGvpE in *Hfx. volcanii* transformants yielded a 13-fold induction of *P_pA_* and an eightfold induction of *P_pD_* when c-*gvpE* was expressed under *P_fdx_* control (Figure 5 and Table 3). However, the final activity of the GvpE-induced *P_pA_* promoter was still lower than the activity of *P_fdx_*.

The *P_pF_* promoter yielded the highest activity in the early exponential growth phase, followed by a continuous reduction up to the stationary growth phase – similar to the growth phase-dependent basal activity of *P_pD_* (Figure 5 and Table 2). Both promoters exhibited the weakest activities of all p-vac promoters tested, but in contrast to *P_pD_*, the *P_pF_* promoter cannot be induced by GvpE. The higher activity of *P_pF_* in the early exponential growth phase underlines the early production of the accessory gas vesicle proteins GvpF through GvpM required in minor amounts during the initial stages of gas vesicle formation (Offner et al., 2000; Winter et al., 2018).

In summary, different temporal activity patterns were observed with the six promoters tested here. All promoters decreased their activity in the stationary growth phase as suggested by the reduced fluorescence of the cells. These results implied that the cells did not accumulate GFP over the time. The relatively slow growth rate of haloarchaea with a generation time of approximately 4 h allows GFP folding and also degradation, which is indispensable for promoter studies.

Overall, mGFP6 is useful to detect weak promoter activities *in vivo* and is even more sensitive compared to the BgaH reporter system. The mGFP6 reporter is also more reliable, since the BgaH activity is influenced by temperature, whereas the fluorescence of GFP is temperature independent. Another advantage of the analysis the mGFP6 fluorescence is that the cells are only washed to remove the media, whereas the BgaH enzyme assay requires cell lysis. Yet, the data obtained with both methods was consistent. The two different haloarchaeal reporter systems now enable the simultaneous and quantitative analysis of two promoters, such as the divergent *P_A_-P_D_* of the gas vesicle protein genes. The BgaH / mRNA analysis applied previously (Marschaus and Pfeifer, 2012) could be substituted by BgaH / GFP to determine both promoter activities in a quantitative way throughout growth in the same cell culture.

### 5′-UTRs Reduced the Translation of *gvp* mRNAs

Many 5′-UTRs of transcripts found in bacteria or eukaryotes supply binding sites for regulatory proteins, small regulatory RNAs, or metabolites (riboswitches) and control the translation. Also, the Shine-Dalgarno sequence is located here, providing the positioning of the mRNA for translation initiation at the 30S ribosomal subunit in bacteria. Thus, sequences and secondary structures of the 5′-UTRs play a pivotal role in gene expression regulation (Cho et al., 2017). In haloarchaea, approximately 30% of the mRNAs are leaderless and lack a 5′-untranslated region (Brenneis et al., 2007). Examples for leaderless transcripts are the *fdx* and the *gvpO* mRNA (Pfeifer et al., 1993; Offner et al., 1996). The *fdx* mRNA occurs in large amounts and is efficiently translated. The insertion of reading frames in the expression vector pJAS35 also results in leaderless transcripts that are efficiently expressed. Also, the lack of the 5′-UTR of the p-*gvpH* mRNA leads to a 15-fold increase in translation (Sartorius-Neef and Pfeifer, 2004). It appears that 5′-UTRs lead to a reduced translation in all these cases.

In this report we used mGFP6 to study the effect of the 5′-UTRs on the expression of p-*gvpACNO* (5′UTR_A_), p-*gvpDE* (5′UTR_D_), and p-*gvpFGHIJKLM* (5′UTR_F_). In all three cases, the deletion of the 5′-UTR resulted in an increase of the GFP fluorescence, especially in early growth stages (Figure 6 and Table 2). Bioinformatic analyses indicate secondary structures in 5′-UTR_A_ and 5′-UTR_D_ that might interfere with the initiation of translation. The higher expression of the leaderless mRNAs could be explained with a higher accessibility of the mRNA to the ribosome. A 1.4- to 2.4-fold increase in fluorescence due to the deletion of 5′-UTR_A_ was observed in the early exponential growth phase, suggesting a growth depending regulation of 5′-UTR_A_ (Figure 6 and Table 2). In contrast, deletion of the 5′-UTR_D_ and 5′-UTR_F_ led to a 3.3- to 4.5-fold higher translation throughout growth when compared to the respective leader-containing transcript.

## Conclusion

Overall, mGFP6 appears to be an easy, fast and sensitive alternative to investigate the gene expression at the level of transcription and translation in haloarchaea. Promoter activities can be determined, and the original vector pPLacZJB18 is useful to substitute *lacZ* with the desired promoter sequences allowing blue/white selection in *E. coli*. mGFP6 is a highly fluorescent, short-lived protein and can be applied to investigate the time-dependent transcription or translation in haloarchaea. In future, the regulatory effect of 5′-UTRs on the *gvp* gene expression will be investigated in further detail.

## Data Availability

The raw data supporting the conclusions of this manuscript will be made available by the authors, without undue reservation, to any qualified researcher.

## Author Contributions

JB and FP planned the study, discussed the results, wrote the manuscript, and approved the final manuscript. JB performed the analysis.

## Conflict of Interest Statement

The authors declare that the research was conducted in the absence of any commercial or financial relationships that could be construed as a potential conflict of interest. The reviewer MLDS declared a past co-authorship with one of the authors FP to the handling Editor.
