# Supplementary material for: Improved GFP Variants to Study Gene Expression in Haloarchaea
Source: Front Microbiol. 2019 May 29;10:1200. doi: 10.3389/fmicb.2019.01200 (PMC6550001; doi:10.3389/fmicb.2019.01200)
Supplement: Supplementary file 1 [file Data_Sheet_1.PDF]

frontiers

Supplementary Material

A Novel GFP Variant to Study Gene Expression in Haloarchaea

Johannes Born and Felicitas Pfeifer*

Microbiology and Archaea, Department of Biology, Technische Universität Darmstadt, Darmstadt, Germany

*** Correspondence:**
Prof. Dr. Felicitas Pfeifer
pfeifer@bio.tu-darmstadt.de

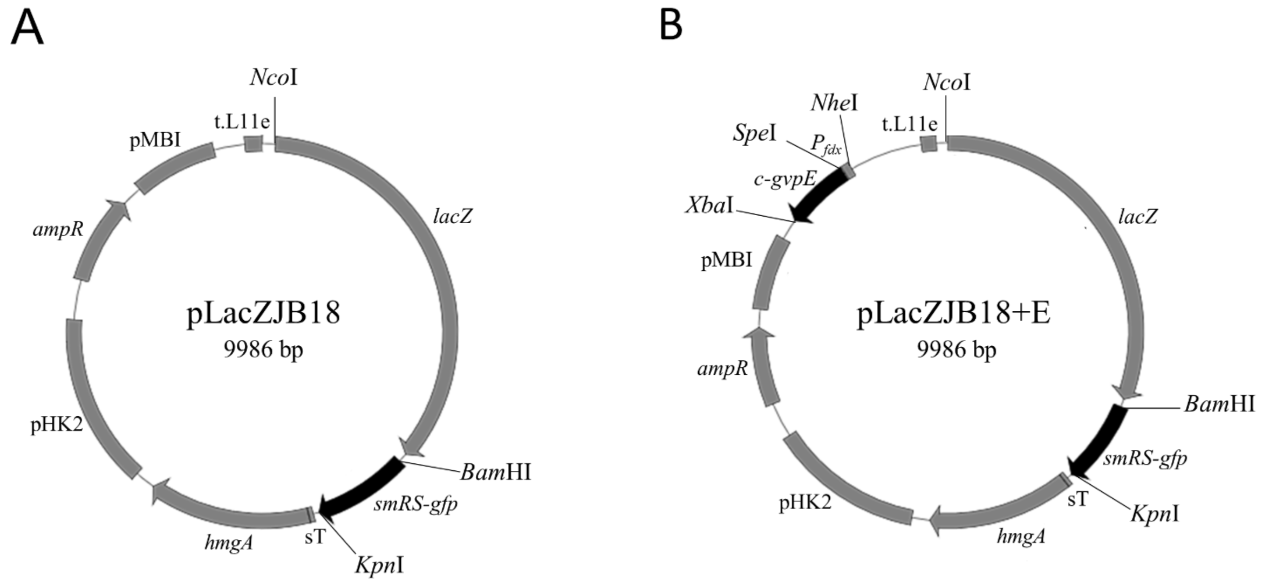

Figure S1. Expression vectors pLacZJB18 **(A)** and pLacZJB18+E **(B)**. Both plasmids contain *lacZ* upstream of the *smRS-gfp* gene. The expression of *gvpE* in pLacZJB18+E is under control of P_{fdx}. For promoter studies *lacZ* was replaced by the regulator sequence *via Nco*I and *Bam*HI, whereas the reporter genes are exchanged using *Bam*HI and *Kpn*I. The promoter-*smRS-gfp* fragment is flanked by an archaeal terminator (t.L11e) and a synthetic terminator.

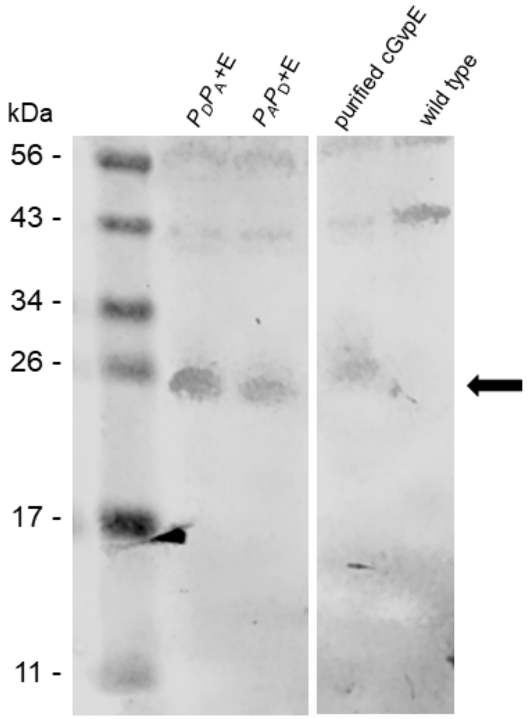

Figure S2. Western analysis performed with the P_DP_A+E and P_AP_D+E transformants. Twenty micrograms of total protein were separated by SDS-PAGE, blotted to a PVDF membrane and incubated with the cGvpE antiserum. The second antibody was tagged with the fluorescent dye IRDye 800 CW (LI-COR) for detection. The image was reversed to black and white. The expected protein mass of cGvpE (21 kDa) is marked by an arrow. Wild type = *Hfx. volcanii* WR340. Marker: Fisher BioReagents™ EZ-Run™ Prestained *Rec* Protein Ladder (fisher scientific).

Table S1. *Haloferax volcanii* strains used.

Strain	Relevant genotype	Reference
WFD11	Deletion of plasmid pHV2	Cline *et al.*, 1989
WR340	His^-	Bitan-Banin *et al.*, 2003
H1424	Δ*pyrE2* Δ*hdrB nph-pitA* Δ*mrr cdc48d-Ct*	Stroud *et al.*, 2012
